# High-intensity treadmill training and self-management for stroke patients undergoing rehabilitation: a feasibility study

**DOI:** 10.1186/s40814-021-00941-w

**Published:** 2021-12-07

**Authors:** Sandra G. Brauer, Suzanne S. Kuys, Jennifer D. Paratz, Louise Ada

**Affiliations:** 1 Discipline of Physiotherapy, School of Health and Rehabilitation Sciences, The University of Queensland, Brisbane, Queensland Australia; 2 School of Physiotherapy, Australian Catholic University, Banyo, Queensland Australia; 3 Burns, Trauma and Critical Care Research Centre, University of Queensland, Brisbane, Queensland Australia; 4 School of Allied Health Sciences, Griffith University, Southport, Queensland Australia; 5 Discipline of Physiotherapy, Faculty of Health Sciences, The University of Sydney, Sydney, Australia

**Keywords:** Stroke, Randomised, Treadmill, Walking, Cardiorespiratory fitness, Physical activity

## Abstract

**Background:**

Physical activity undertaken by stroke survivors is generally low. This trial investigated the feasibility of delivering a high-intensity treadmill and self-management program to people with stroke undergoing inpatient rehabilitation and determine whether physical activity, walking ability and cardiorespiratory fitness could be increased.

**Method:**

A phase I, single-group, pre-post intervention study was conducted with stroke survivors undergoing inpatient rehabilitation who could walk. Participants undertook a high-intensity treadmill and self-management program for up to 30 min, three times a week for 8 weeks under the supervision of their usual physiotherapist. Feasibility was determined by examining compliance, satisfaction and adverse events. Clinical outcomes were amount of physical activity, walking ability, and cardiorespiratory fitness collected pre-training (week 0), post-training (week 8), and at follow-up (week 26).

**Results:**

Forty stroke survivors participated, completing 10 (SD 6) sessions, 94% at the specified training intensity, with high satisfaction and no adverse events related to the intervention. At week 8, participants completed 2749 steps/day (95% CI 933 to 4564) more physical activity than at week 0. Walking distance increased by 110 m (95% CI 23 to 196), walking speed by 0.24 m/s (95% CI 0.05 to 0.42), and VO2 peak by 0.29 ml/kg/min (95% CI 0.03 to 0.56). At week 26, increases in physical activity, walking distance and speed, and cardiorespiratory fitness were maintained.

**Conclusions:**

A high-intensity treadmill training program embedded within a self-management approach during inpatient rehabilitation appears feasible and potentially may offer sustained improvements in physical activity, walking ability, fitness, and quality of life. A randomised trial is warranted.

**Trial registration:**

This feasibility study was registered with the Australian New Zealand Clinical Trials Registry (ACTRN12613000764730).

## Key messages on feasibility

- Prior to this study, high-intensity treadmill training had not been embedded in a self-management approach during stroke rehabilitation to enable a transition to sustainable physical activity level follow discharge
- A high-intensity treadmill training program embedded within a self-management approach during inpatient stroke rehabilitation appears feasible
- Given this approach may offer sustained improvements in physical activity, a randomised trial is warranted.

## Background

Stroke survivors, regardless of type, have at least three times the risk of further cardiovascular events than the general population [1–3]. There is strong evidence that physical activity has a protective effect against stroke [4, 5] and that not undertaking enough physical activity increases the risk of recurrent stroke [6, 7]. The level of physical activity undertaken by stroke survivors living in the community is generally low [8–11]; less than half that of age-matched adults [12, 13]. However, increasing the level of physical activity after stroke is challenging, perhaps because multiple factors contribute. For example, physical activity is correlated with poor reduced walking ability [14], cardiorespiratory fitness [15], and low self-efficacy [16].

Clinical guidelines suggest that for stroke survivors who experience walking difficulties treadmill training should be provided [17]. High-intensity treadmill training is an intervention that targets walking difficulty and reduced cardiorespiratory fitness. Using high-intensity treadmill training, i.e., training to a target heart rate around 40–60% of heart rate reserve, has been shown to increase cardiorespiratory fitness in chronic stroke [18, 19]. However, this type of training is not usually started during inpatient rehabilitation [19]. Commencing rehabilitation as early as possible has been recommended [17] particularly as the majority of motor recovery occurs in the first few months after stroke [20]. Treadmill walking has been shown to have a higher cardiovascular demand than usual rehabilitation interventions and is safe for people early after stroke [17, 21]. In a pilot study, we have shown that high-intensity treadmill training during inpatient rehabilitation is feasible, is not detrimental to the walking pattern [22, 23], and may result in an improvement in walking ability [24]. However, we have not shown that it increases cardiorespiratory fitness.

Furthermore, one of the main findings of studies investigating interventions to cardiorespiratory fitness, is that the benefit does not last beyond the period of intervention [25]. It is therefore important to support stroke survivors to exercise independently in the long term [26]. Exercise behaviours after stroke are predicted by exercise self-efficacy and outcome expectations [27, 28] thus should be addressed. Self-management approaches such as the health action plan approach [29] addresses these issues and have been shown to lead to lasting changes in activity levels in other chronic diseases [30], but has not been investigated in stroke. It appears that a comprehensive, patient-centered, goal-oriented approach to lifestyle modification is required early in rehabilitation that addresses both the ability for stroke survivors to be active, and the motivation to do so. Maintaining high levels of physical activity via improved walking ability and ongoing cardiorespiratory fitness should also confer a range of benefits, such as a decrease in cardiovascular risk [31] depression and burden of care as well as an improvement in self-efficacy, participation, and quality of life.

The aim of this Phase I intervention study, therefore, was to investigate the feasibility of, and potential for, self-management to increase physical activity via an increase in self-efficacy in stroke survivors with mild disability. The specific research questions were:

1. Is it feasible to deliver a high-intensity treadmill embedded in a self-management approach to people with stroke undergoing inpatient rehabilitation and to determine whether it can increase physical activity, walking ability, and cardiorespiratory fitness?
2. Can self-management increase physical activity, walking ability and cardiorespiratory fitness in the short term (2 months) as well as self-efficacy of walking, health-related quality of life, participation and self-reported physical activity in the longer term (6 months)?

We hypothesised it would be feasible to deliver a high-intensity treadmill embedded in a self-management approach to people with stroke undergoing inpatient rehabilitation to increase physical activity, walking ability, and cardiorespiratory fitness, without incurring adverse events. Additionally, we hypothesised that self-efficacy of walking, health-related quality of life, participation, and self-reported physical activity benefits would be sustained over 6 months.

## Method

### Design

**Fig. 1:**
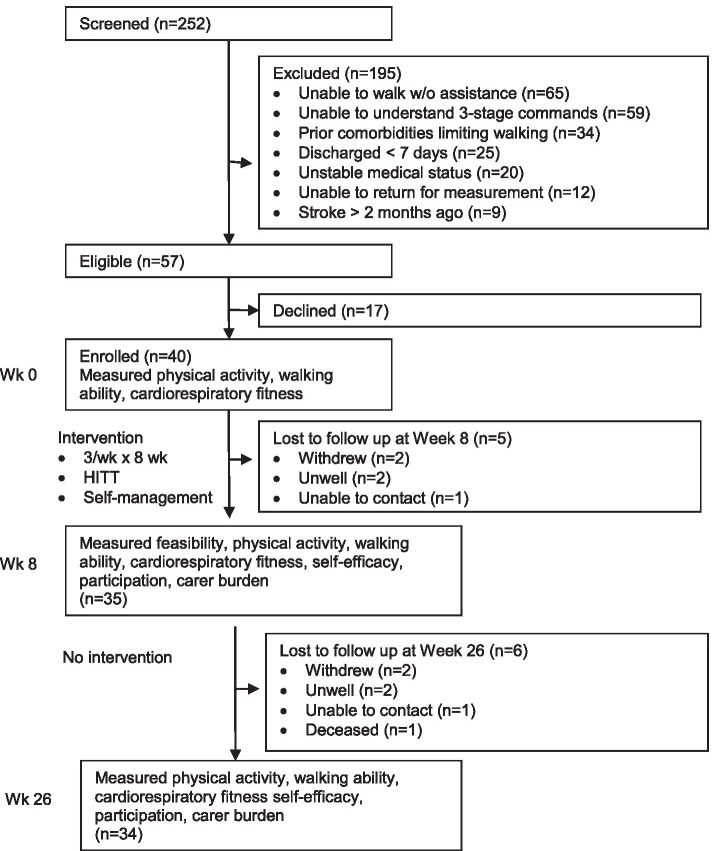
Design and flow of participants through the study

A phase I, single-group, pre–post intervention study was carried out (Fig. 1). Stroke survivors undergoing rehabilitation were recruited from two public inpatient rehabilitation units in Australia. They undertook a high-intensity treadmill and self-management program for up to 30 min, three times a week for 8 weeks under the supervision of their usual physiotherapists. Measures were collected at baseline, at 2 months, and at 6 months by an independent assessor. The study obtained ethical approval from hospital and university Human Research Ethics Committees and was registered on the Australian New Zealand Clinical Trials Registry (ACTRN12613000764730).

### Participants

Individuals with stroke undergoing inpatient rehabilitation were screened and invited to participate if they were within 2 months of a stroke (confirmed by CT scan or clinical diagnosis); aged 18 years or older; able to walk 10 m independently (without physical assistance but with or without an aid); and able to follow three-stage commands. Individuals with stroke were excluded if they were unable to walk independently prior to current stroke, had co-morbidities relating to the lower limbs that might limit their walking (such as other neurological conditions and osteoarthritis), had unstable cardiac status (which would preclude them from participating in intensive training as deemed by treating medical team), were unable to provide informed consent, live too far away to return for post-intervention assessments, or had an anticipated length of stay of < 1 week. All stroke survivors were eligible for inclusion regardless of stroke type. Information was collected to describe the sample included: age, sex, description of the stroke (time, side affected, type, location, severity), aphasia, thrombolysis, co-morbidities, and previous living arrangements.

### Intervention

The intervention consisted of a program of high-intensity treadmill training plus self-management. The program was performed for up to 30 min, three times a week for up to 8 weeks. The intervention was carried out across inpatient and outpatient departments. The program was performed with the supervision of the usual physiotherapist and replaced a similar period of time of gait training. Usual multi-disciplinary inpatient rehabilitation consisting of task specific training.

The high-intensity treadmill training was based on the minimum requirements for training cardiorespiratory fitness (6). This consisted of up to 30 min walking on a treadmill at an intensity between 40 and 60% heart rate reserve or a Borg Rating of Perceived Exertion of 11–14 [32], and participants were encouraged to hold the handrail. Target heart rates were calculated according to the Karvonen method [33] adjusting for beta-blockers. The use of a harness support system including body weight support was available at both participating facilities and could be used at the discretion of the treating physiotherapist. Treating physiotherapists could provide rest breaks as required.

The self-management approach was based on the Health Action Process Approach (HAPA) [29] and Goal Setting Theory [34]. It consisted of 5–10-min sessions, three times a week for up to 8 weeks. The sessions were delivered prior to or during the treadmill sessions by the treating physiotherapist. A pragmatic, individualised, patient-centred approach was taken whereby participants were educated and coached in goal setting, planning, and adaptation of exercise programs focussed on goals related to increasing and maintaining physical activity [35]. A variety of strategies to increase and monitor activity were discussed with participants including formal programs (e.g. walking groups), self-modified exercise programs, exercise buddies, and the use of activity monitoring devices such as pedometers.

Physiotherapists delivering the experimental intervention were trained in high-intensity treadmill training and self-management education via in-services (*n* = 4), provision of workbooks, and discussion sessions with new staff and/or students working in the area. Physiotherapists delivering the intervention were the treating physiotherapists. Physiotherapy students supervised by treating physiotherapists were also trained in the delivery of the intervention. Workbooks for the physiotherapists and patients were provided that included educative material, progressions of both treadmill and self-management over 8 weeks, and opportunities for recording, planning, and reflecting on physical activity.

### Outcome measures

#### Feasibility

Feasibility of the program was determined by examining recruitment and intervention compliance, adverse events and satisfaction. Feasibility of recruitment was recorded as the proportion of people in rehabilitation that were suitable for this intervention and agreed to participate. Compliance with the program was recorded as the number of sessions completed, the parameters of treadmill training achieved (e.g. time spent in training, intensity of training) and self-management components (e.g. provision of resources, risk perception, outcome expectancy, task self-efficacy, maintenance self-efficacy, action planning, and coping planning). Intervention fidelity was checked through session audits of treadmill and self-management components. Adverse events (serious, e.g. hip fracture and minor, e.g. fatigue) were recorded. Participant satisfaction with the intervention was assessed using a 5-point Likert scale questionnaire (strongly agree to strongly disagree) asking about the treadmill and self-management components of the intervention.

#### Clinical outcomes

Physical activity walking ability and cardiorespiratory fitness were measured at week 0 (pre-training), 8 (post-training), and 26 (follow-up). Physical activity was measured with an accelerometer (ActivPAL) that measured number of steps/day over four days. The accelerometer was placed on the anterior thigh of the unaffected side. This device has been shown to be accurate in recording activity including steps in people following stroke walking at varied speeds, indoors, and out [36]. Walking ability measures included the 6-min Walk Test, a clinical measure of endurance, which was conducted according to the American Thoracic Society guidelines [37] and reported as distance walked as well as the 10-m Walk Test [38] which was conducted at preferred speed over 14 m using usual gait aids, and reported as mean gait speed. Cardiorespiratory fitness (VO_2_peak) was recorded during the 6-min Walk Test by a fully portable compact metabolic system using breath by breath analysis (Metamax 3B (Cortex, Biophysik, Germany).

Self-efficacy of walking, health-related quality of life, participation, and perception of physical activity were collected at weeks 8 and 26. Self-efficacy of walking ability was measured using the Ambulatory Self-Confidence Questionnaire [39]. Health-related quality of life was measured using the EuroQual-5D [40] across five domains and overall health state using a visual analogue scale. Participation was measured using the Impact on Participation and Autonomy Questionnaire [41] in the domains of indoor and outdoor autonomy, family role, social relations, work, and education. Perception of physical activity was measured using the Physical Activity Scale for Individuals with Physical Disabilities in the domains of home repair, lawn and garden work, housework, vigorous sport and recreation, moderate sport and recreation, and occupation [42].

### Sample size

A sample size calculation is not required for a feasibility study [43]. The sample of 40 was one primarily of convenience aiming to recruit people with a range of ages, stroke type and severity, sex, and walking ability post-stroke.

### Data analysis

Descriptive statistics were used to describe the sample and report on outcomes of feasibility. Within-group differences of clinical outcomes between week 0 and 8, 0 and 26, were analysed using linear mixed effects models to avoid casewise deletion due to missing data. Within-group differences of clinical outcomes between week 8 and 26 were analysed using paired *t* tests. SPSS Version 22.0 (IBM Corp, Armonk, NY) was used, with a *p* value of 0.05 set as the level of significance.

## Results

### Characteristics of participants

Forty participants were recruited to this study. Participant characteristics are presented in Table 1.

**Table 1:** Baseline characteristics of participants and therapists

Characteristic	
Participants	*n* = 40
Age (years), mean (SD)	68 (13)
Sex, *n* males (%)	27 (64)
Time since stroke (*days*), mean (SD)	27 (24)
Side of hemiplegia, *n* right (%)	26 (62)
Type of stroke, *n* ischemic (%)	34 (81)
Location of stroke, *n* (%)	
TACI	2 (5)
PACI	29 (69)
POCI	6 (14)
LACI	3 (7)
Modified Rankin scale, *0–5*, median (IQR)	3 (3–3)
Aphasia, *n* yes (%)	8 (19)
Thrombolysis, *n* yes (%)	3 (7)
Co-morbidities (number), mean (SD)	3.0 (1.8)
Previous living arrangements, *n* alone (%)	13 (31)
Therapists	*n* = 12
Experience, years, mean (SD)	10 (8)
Sex, number male (%)	4 (33)
Participants treated, mean (SD)	8 (13)

*TACI* total anterior circulation infarct, *PACI* partial anterior circulation infarct, *POCI* posterior circulation infarct, *LACI* lacunar circulation infarct

### Feasibility

Over 21 months, 252 people were screened, of which 73% were excluded. The main reasons for exclusion were an inability to walk (33%) or follow three stage commands (30%). Sixty-nine people (27%) were eligible. Of these, 58% participated (*n* = 40), 25% refused (*n* = 17), and 17% could not meet study requirements being unable to return for assessments (Fig. 1). Nine people were excluded as having a stroke > 2 months ago though met all other eligibility criteria. Retention of participants at week 8 was 88% and 85% at week 26.

Participants completed on average 10 (SD 6) sessions over 17 (SD 1) days, averaging 17 (SD 2) min/session on the treadmill alone, i.e. 25% of possible training. Heart rate monitors were not available for all participants for all sessions and intervention intensity was measured using the Borg scale of perceived exertion. Compliance with the intensity of the treadmill training was high, with 94% of completed sessions carried out at the intended intensity (Borg > 11/20). Two participants completed two sessions each below the required intensity. Self-management components were inconsistently reported.

Fidelity checking observed accurate reporting of treadmill duration and intensity recorded using Borg. Task self-efficacy was the most common self-management component; discussed in 88% of the audited sessions, initiated by participants 28% of the time and by physiotherapists 88% of the time. Outcome expectance (22%) and action planning (19%) were next most frequently discussed components of self-management. Risk perception and coping planning were infrequently observed. There were four serious adverse events (1 death, 2 lower limb fractures, 1 brain tumour), and all were unrelated to the intervention or trial. No minor adverse events occurred. Participant satisfaction levels were high, with 85% agreeing or strongly agreeing that they felt confident walking on the treadmill and felt it improved their walking. Similarly, 73% agreed or strongly agreed that discussions with the trainer helped them to overcome barriers to exercise and 93% agreed or strongly agreed that they felt confident that they would live an active lifestyle in the future (Table 2).

**Table 2:** Percentage of participants that strongly agree or agree with questions about their satisfaction with the intervention

Question	Strongly agree/agree (*n* = 24)
Treadmill component	
I liked walking on the treadmill	70%
I found walking on the treadmill to be challenging	45%
Walking on the treadmill helped improve my ability to walk	85%
I felt confident during treadmill training	85%
Treadmill training was difficult	40%
I experienced pain during treadmill training	10%
I experienced mental tiredness/ fatigue during treadmill training	40%
I experienced physical tiredness/ fatigue during treadmill training	60%
I was anxious during treadmill training	15%
Self-management component	
Talking to my trainer about goal setting helped me to set personal goals related to physical activity	73%
My trainer helped me to develop coping strategies to overcome barriers to being physically active	87%
The discussions I had with my trainer have helped me to think about physical activity in a positive way	93%
I feel confident that I will continue to live an active lifestyle now that the treadmill training has finished	47%

### Clinical outcomes

Clinical outcomes are presented in Tables 3 and 4. At week 8, participants completed 2749 steps/day (95% CI 933 to 4564) more physical activity than at week 0. They had increased walking distance by 110 m (95% CI 23 to 196), walking speed by 0.24 m/s (95% CI 0.05 to 0.42), and VO_2_ peak by 2.2 ml/kg/min (95% CI − 0.2 to 4.5). At week 26, increases in physical activity, walking distance and speed, and cardiorespiratory fitness were maintained (Table 3). The portable metabolic system was not available when the study commenced resulting in only 14 participants recording baseline VO_2_ peak.

**Table 3:** Mean (SD) outcomes at week 0, 8, 26 and mean (95% CI) difference between time points

Outcome	Times			Difference between times**	
	Week 0 (*n* = 40)	Week 8 (*n* = 35)	Week 26 (*n* = 34)	Week 8 minus week 0	Week 26 minus week 0
Physical activity Accelerometry *(steps/day)*	3024 (1955)	5773 (2943)	6664 (3371)	2749 (933 to 4564)	3640 (1734 to 5545)
Walking distance 6-min Walk Test *(m)*	277 (142)	368 (144)	402 (141)	110 (23 to 196)	125 (40 to 210)
Walking speed 10-m Walk Test *(m/s)*	0.72 (0.29)	0.99 (0.36)	1.01 (0.33)	0.24 (0.05 to 0.42)	0.33 (0.17 to 0.50)
Cardiorespiratory fitness* VO_2_ peak *(ml/kg/min)*	11.0 (3.3) *n* = 14	13.6 (3.3) *n* = 20	14.9 (4.1) *n* = 24	2.2 (− 0.2 to 4.5) *n* = 6	4.7 (1.8 to 7.5) *n* = 6

^*^Missing VO2 data due to unavailability of metabolic system

**Differences between times are the average of paired differences

**Table 4:** Mean (SD) outcome measures at week 8 and 26 and mean (95% CI) difference between time points

Outcome	Times		Difference between times**
	Week 8 (*n* = 35)	Week 26 (*n* = 34)	Week 26 minus Week 8 (*n* = 34)
Self-efficacy of walking ASCQ *(0 to 10)*	8.3 (1.6)	8.6 (1.5)	0.5 (− 0.1 to 1.0)
Quality of Life EuroQual-5D VAS *(0 to 100)*	65 (17)	74 (15)	9 (2 to 17)
Participation IPAQ *(30 to 155)*	18 (17)	17 (18)	− 4 (− 10 to 2)
Perception of physical activity PASIPD *(0 to 100)*	7 (5)	9 (7)	2 (− 3 to 7)

*ASCQ* Ambulatory Self Confidence Questionnaire, *IPAQ* Impact on Participation and Autonomy Questionnaire, *PASIPD* Physical Activity Scale for Individuals with Physical Disabilities

**Differences between times are the average of paired differences

At week 26, participants had a 9% (95% CI 2 to 17) higher quality of life than at week 8. There were no statistically significant differences in self-efficacy of walking, participation, or perception of physical activity (Table 4).

## Discussion

This intervention of high-intensity treadmill training embedded in a self-management approach appears, at least in part, feasible. Approximately 30% of inpatients screened for participation in this study met the eligibility criteria and included stroke survivors with varying stroke severity and type. Physiotherapists delivering the intervention were able to meet the target duration and intensity of the high-intensity treadmill training sessions and include components of self-management in discussions with participants. However, participants received only a quarter of the planned number of treadmill sessions. Participants were satisfied with the intervention and few adverse events occurred; none associated with delivery of the intervention. Physical activity levels, walking ability, and cardiorespiratory fitness all improved immediately following the intervention and these improvements were maintained beyond the intervention. Some modifications to this intervention are needed for further investigation.

This intervention of high-intensity treadmill training embedded in a self-management approach appears suitable for a range of stroke survivors. Participants comprised stroke survivors with a variety of types and locations of stroke, and varying motor abilities. Recruiting stroke survivors to research projects is challenging with many trials either not meeting the target sample size or extending the recruitment time period in an attempt to meet the target sample size [44]. Commonly cited issues regarding recruitment include stroke-related impairments [45], delayed ethical or site approval and trial staff appointments [44], and slow recruitment rates due to patient eligibility, agreement to participate, or staffing issues [44–46]. This trial was not without these issues, and only recruited two-thirds of our planned sample but was still successful in recruiting approximately one-third of all stroke survivors admitted to the participating units. One potential contributor to our recruitment success was probably an open research design where participants know the intervention they will be receiving. This has been shown to optimise recruitment [47]. Another contributor to our recruitment success may have been that participants liked the intervention or the opportunity for increased rehabilitation. This has not been explored in previous research examining recruitment [44, 45, 47] but our satisfaction data appear to support this hypothesis. Recruitment for this trial ceased when funding ran out. The sample size recruited was similar to our previous pilot study recruiting 30 sub-acute stroke survivors which was sufficient to demonstrate improvements in walking outcomes [24] and is in line with the median sample size of feasibility trials [48].

The dose of intervention was only partially delivered. We had intended to deliver 24 sessions of up to 30 min per session and at a high intensity. On average, 10 sessions were delivered, for approximately 17 min per session, at a high intensity. The intervention was designed to be delivered to participants during the transition from inpatient rehabilitation to living in the community. For many participants, there were limited opportunities to continue the intervention as intended once discharged from inpatient rehabilitation. Participants largely received little or no ongoing outpatient rehabilitation at the participating facilities. Some participants were referred to community-based rehabilitation but these services did not offer high-intensity treadmill training. While the dose of intervention was low, the intensity was delivered as planned. Intensity was planned to be monitored using heart rate. Chest strap type monitors were not available for all participants. However, Borg rating scale was consistently used to monitor intensity and has been shown to be valid for people following stroke to monitor intensity up to 70% peak oxygen uptake [49], though future studies should aim to monitor heart rate directly. Few adverse events occurred, despite therapist concerns that high-intensity training may adversely impact walking pattern. In a future trial, the ability to follow patients from inpatient to outpatient/community rehabilitation in order to deliver a sufficient dose needs to be considered.

Participants almost doubled their step count per day, and most importantly, this increase was maintained at 6 months. Similarly, systematic reviews have found immediate improvement in walking speed [50] and both immediate and long term improvement in walking speed and distance [51] following a treadmill training intervention in ambulatory stroke. There is no such evidence for physical activity [28]. It is reasonable to suggest that increased physical activity would be anticipated as participants transitioned from inpatient rehabilitation to living in the community and may not be due to our intervention alone. Regardless, physical activity levels of stroke survivors are known to be low [8–11], further investigation is needed on the impact of treadmill training on physical activity levels in stroke survivors. Although peak oxygen consumption also improved, these results should be interpreted with caution due to the small sample size in this outcome.

These findings are encouraging for self-management in physiotherapy rehabilitation. Increasing pressure for early discharge following stroke, shorter length of stay [52] coupled with more severe disability [53] will influence rehabilitation and outcomes of stroke survivors. Self-management offers a potential strategy to equip stroke survivors with the capabilities to drive their ongoing recovery. Self-management is an individualised intervention which was able to be delivered by treating physiotherapists. Task self-efficacy was the most common component. Self-efficacy has been shown to be important following stroke; associated with quality of life, depression, and activities of daily living [54]. However, the best approach and delivery of self-management has yet to be determined [55]. Self-management to improve physical activity of stroke survivors is less clear with a recent systematic review unable to demonstrate benefit [56]. Future research needs to further explore how self-management is delivered and identify which components may be beneficial to improving physical activity, both in the immediate and long-term participation in health promoting physical activity levels. Stroke survivors in the current study liked the treadmill intervention and felt confident to complete the training. Additionally, stroke survivors found engaging with the physiotherapist delivering the intervention helped them to think more positively about engaging in physical activity. However, participants were not confident to continue a physically active lifestyle once the training finished. This study supports the potential of embedding self-management in a comprehensive intervention for improving physical activity, though components which are likely to contribute to long-term participation in physical activity remain unclear.

This study is not without its limitations. Our sample was one of convenience and the study was conducted in two rehabilitation facilities in Queensland, Australia. These findings may not be generalisable to other settings and across the broad stroke survivor population. A number of participants (15%) were unable to return for follow-up measurements, potentially biasing our findings. It is important to note that this high-intensity treadmill training intervention embedded in a self-management approach had some challenges to being delivered by treating physiotherapists. Difficulties were noted with completing the required number and duration of treadmill sessions particularly during the transition to living in the community, as well as recording self-management and measuring oxygen uptake.

## Conclusions

In summary, delivering the high-intensity treadmill training embedded in a self-management approach appears to be feasible for stroke survivors during inpatient rehabilitation. The clinical outcomes of physical activity and walking measured in this feasibility trail suggest this intervention has the potential to be beneficial. Therefore, a randomised controlled trial is warranted. However, some modifications appear necessary including the need to be able to deliver the intervention across the transition from inpatient to outpatient settings, self-management needs to be more structured and recording of components needs further consideration as well as a dedicated metabolic cart is required to optimise measurement of oxygen uptake.

## Data Availability

Datasets used and analysed during the current study are available from the corresponding author on reasonable request.

## Abbreviations

HAPA: Health Action Process Approach
VO_2_ peak: Peak oxygen uptake
